# The Brain–Heart Link: A Case Report of a Critically Located Multiple Sclerosis Lesion in the Brainstem Leading to Recurrent Takotsubo Syndrome

**DOI:** 10.3389/fcvm.2021.674118

**Published:** 2021-08-09

**Authors:** Florentijn Risseeuw, Pegah Masrori, Ingrid Baar, Simon Nicolay, Constantijn Franssen, Barbara Willekens

**Affiliations:** ^1^Department of Cardiology, Antwerp University Hospital, Edegem, Belgium; ^2^Department of Neurology, Antwerp University Hospital, Edegem, Belgium; ^3^Department of Intensive Care Medicine, Antwerp University Hospital, Edegem, Belgium; ^4^Department of Radiology, Antwerp University Hospital, Edegem, Belgium; ^5^Cardiovascular Diseases, Genetics, Pharmacology and Physiopathology of Heart, Blood Vessels and Skeleton (GENCOR), University of Antwerp, Wilrijk, Belgium; ^6^Translational Neurosciences, Faculty of Medicine and Health Sciences, University of Antwerp, Wilrijk, Belgium

**Keywords:** multiple sclerosis, brainstem, autonomic dysfunction, case report, Takotsubo syndrome, stress cardiomyopathy

## Abstract

Various central nervous system (CNS) diseases, including neurovascular and neuroinflammatory diseases, can lead to stress cardiomyopathy, also known as Takotsubo syndrome (TTS). We present a case of a 69-year-old woman with cardiovascular comorbidities, suffering from repeated episodes of TTS and respiratory failure due to a critical lesion in the brainstem, leading to a diagnosis of multiple sclerosis (MS). Despite aggressive treatment, intractable and recurrent symptoms in our patient occurred. Repeated bouts of autonomic dysfunction and respiratory failure ultimately led to installment of palliative care and the patient passing away. TTS should raise suspicion for underlying neurological diseases. Thorough questioning of previous neurological symptoms and extensive neurological workup is warranted. MS should be considered as a trigger of TTS also in elderly patients with cardiovascular risk factors.

## Introduction

Stress cardiomyopathy, also known as Takotsubo syndrome (TTS) or broken-heart syndrome (1, 2), can be caused by acute or chronic central nervous system (CNS) diseases, including subarachnoid or intracerebral hemorrhage, epilepsy, ischemic stroke, migraine, encephalitis, traumatic brain injury, posterior reversible encephalopathy syndrome (PRES), and amyotrophic lateral sclerosis (ALS) (3–5). Both classic TTS and inverted TTS have also been described in patients known to suffer from multiple sclerosis (MS) (3, 4, 6–19). We report a case of recurrent classic TTS in combination with episodes of respiratory failure and other signs of autonomic dysfunction, which led to a diagnosis of MS in an elderly woman with cardiovascular risk factors.

## Case Description

**Figure 1:**
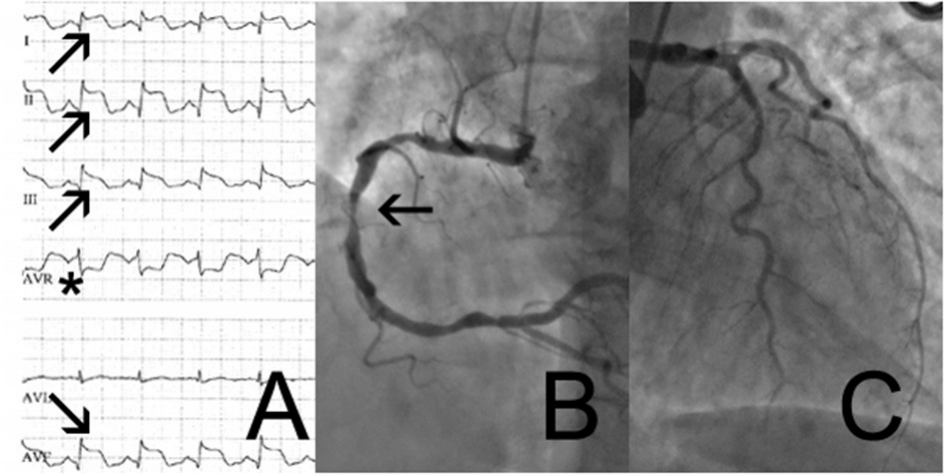
6-lead monitor ECG **(A)** with ST-elevation in the inferolateral leads I,II,III,aVF (arrows) and ST depression in aVR (asterix). Coronary angiogram showing right coronary artery **(B)** with significant stenosis of the mid section of the right coronary artery (arrow) and coronary atheromatosis with not significant coronary artery disease of the left circumflex- and left anterior artery **(C)**.

**Figure 2:**
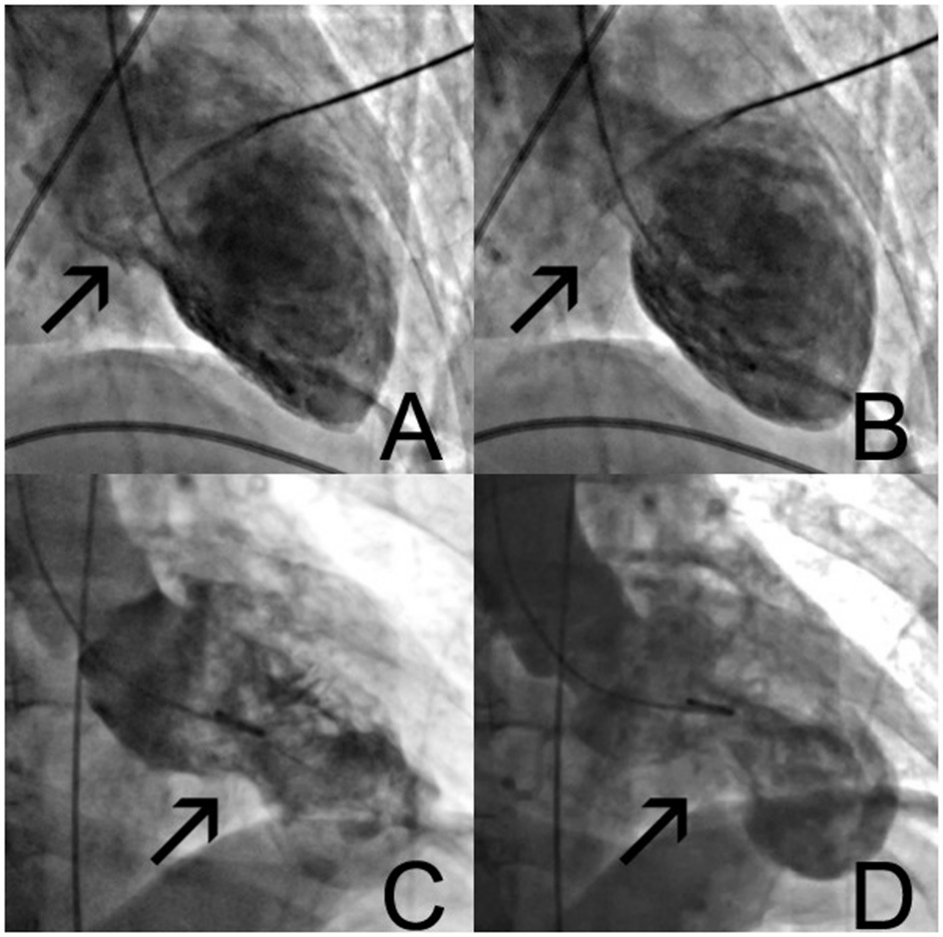
Left ventricular ventriculography **(A–D)**. In normal diastole **(A)**, with typical apical ballooning and contraction of only the basal parts of the ventricle **(B)**. Control angiogram after readmission 7 days later **(C)**, showing different morphologies with mid-section hypercontraction and apical sparing **(D)**. See also Supplementary Data Clips 1, 2.

A 69-year-old woman with a history of cigarette smoking, arterial hypertension, type 2 diabetes mellitus, hypercholesterolemia, right subcapital femur fracture, and possible stroke was transferred to our institution for coronary angiography (CAG) because of an episode of thoracic discomfort and persistent dyspnea. The working diagnosis was inferolateral ST-segment elevation myocardial infarction (STEMI). The provided print of 6-lead electrocardiogram (ECG) monitor strip showed inferolateral STEMI (Figure 1A). On admission, she had a blood pressure of 100/70 mmHg, heart rate of 110 beats per minute, and peripheral oxygen saturation of 100% with 2 L of oxygen. Tachypnea, hyperhidrosis, bilateral rhonchi, and right-sided facial palsy were present on physical examination. Biochemical findings included elevated cardiac troponin I (peak level: 5.68 μg/L, normal value <0.045 μg/L; day 1, 5.21 μg/ml; day 2 morning, 4.74 μg/ml; day 2 evening 5.68 μg/ml) and creatine kinase-MB (CK-MB) (peak level: 32.6 μg/L, normal value <3.6 μg/L). Natriuretic peptide levels were not assessed at any timepoint. Chest radiograph was normal. CAG showed mild (50%) stenosis of the mid left anterior descending artery and a significant (70%) stenosis of the right coronary artery (RCA) (Figures 1B,C). A 12-lead ECG showed slight residual ST elevation in the infero-anterolateral leads with Q wave formation in the same region (Supplementary File 12-lead ECG 1). Ventriculography revealed apical ballooning (Figures 2A,B) with a decreased left ventricular ejection fraction (LVEF) of 33%. She was diagnosed with TTS, although typical (emotional) triggers were absent.

Besides thoracic complaints, the patient mentioned blurred vision, double vision, and dysphagia, which had been present for more than 2 weeks prior to admission. She had experienced an episode of gait imbalance and right-sided facial palsy attributed to a possible stroke 4 months earlier. Her current neurological symptoms were attributed to a new stroke after excluding hemorrhage on a brain computed tomography (CT) scan, which was completely normal, without signs of recent or old brain infarction. Eight days later, she underwent uncomplicated percutaneous coronary intervention (PCI) with drug-eluting stent (DRES) implantation of the coronary artery stenosis of the RCA.

Seven days after PCI, she was readmitted to the intensive care unit (ICU) due to cardiogenic shock and respiratory failure. Supportive treatment with IV dobutamine, switched to IV noradrenaline after 2 days, and mechanical ventilation was started. ECG revealed non-STEMI with non-significant ST elevation and biphasic T waves in anterior leads (Supplementary File 12-lead ECG 2). Serum troponin I levels remained low (0.118 μg/ml for 2 days in a row). CAG did not demonstrate restenosis of the proximal RCA at the level of the DRES. Ventriculography revealed apical ballooning of the left ventricle with hyperdynamic midsegments, compatible with TTS with a different morphology than at first presentation (Figures 2C,D). This was confirmed by transesophageal echocardiography (TEE). Intracardiac thrombi were absent. TTS resolved in the next days, and cardiac function improved with residual mild hypokinesia of the left ventricle and an LVEF of 53% with evolving deep negative T waves on ECG (Supplementary File 12-lead ECG 3).

Several episodes of acute arterial hypertension (maximum 220 mmHg systolic) occurred, accompanied by respiratory failure with hypoventilation and hypercapnia necessitating invasive ventilation and treatment for hypertension with IV nicardipine. Episodes of brady- and tachycardia occurred, and profound sweating despite hypothermia (lowest temperature 33.5°C) was present. Two days after cessation of IV noradrenaline, plasma catecholamines were measured: metanephrine level was normal (87 pg/ml, reference value 90 pg/ml), but normetanephrine was increased (949 pg/ml, reference value 200 pg/ml). The 24-h urinary excretion of adrenaline, dopamine, and metanephrine was normal. Excreted levels of norepinephrine (416 μg/24 h, reference values 14–50) and normetanephrine (1,709 μg/24 h, reference value upper limit 769) were increased in urine. CT of thorax and abdomen did not show lesions suggestive of paraganglioma nor pheochromocytoma. Iodine-123-labeled MIBG scintigraphy was normal.

**Figure 3:**
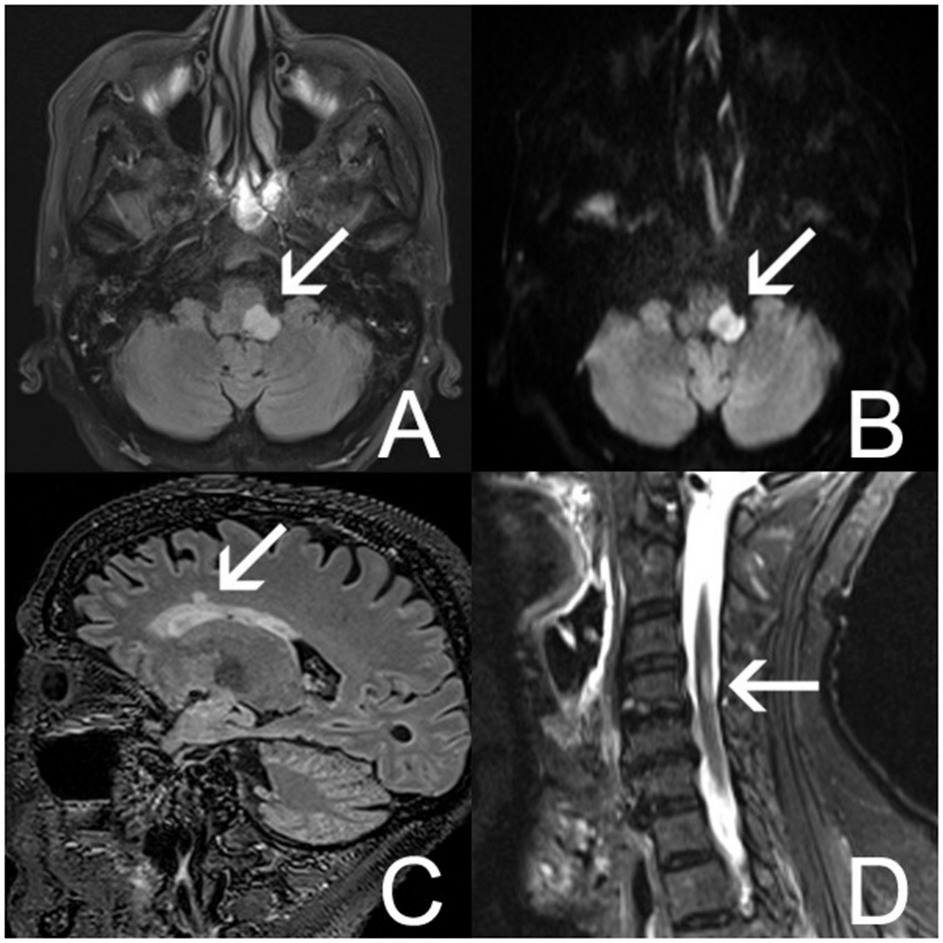
Brain MRI **(A–D)**. **(A)** Axial fluid-attenuated inversion recovery (FLAIR) MRI image shows a well-demarcated, hyperintense lesion in the left posterolateral part of the medulla oblongata (arrow). **(B)** Axial diffusion-weighted imaging (DWI) shows restriction of diffusion in this lesion (arrow). **(C)** Sagittal FLAIR MRI image shows a nodular hyperintensity in the right frontal lobe (arrow). **(D)** Sagittal short tau inversion recovery (STIR) image shows a hyperintense lesion in the cervical medulla at C4 (arrow).

Neurological evaluation showed diplopia, right-sided hemiparesis, a bilateral pyramidal syndrome, and Cheyne–Stokes breathing pattern. An underlying CNS disease triggering TTS and autonomic dysregulation was suspected. Electroencephalography showed no epileptic activity. Brain magnetic resonance imaging (MRI) showed a well-demarcated, oval-shaped T2 hyperintense lesion with diffusion restriction in the left cerebellar peduncle extending to the posterolateral part of the medulla oblongata (Figures 3A,B). Other T2 hyperintense lesions were found in the pons, in the left temporal and right frontal periventricular white matter, and in the cervical spinal cord at the level of C4 (Figures 3C,D). One juxtacortical T2 hyperintense lesion was found in the left parietal lobe. In the differential diagnosis, systemic autoimmune diseases and neuroinflammatory diseases such as MS and neuromyelitis optica were considered. Infectious and autoimmune diseases other than MS were excluded via laboratory tests. Bedside fundoscopy was normal without signs of vasculitis. Due to the need for dual antiaggregant therapy after recent PCI of RCA, lumbar puncture was not performed.

In retrospect, 33 years prior, two episodes of spontaneously remitting neurological symptoms occurred with an interval of 2 months. MRI of the cervical spine, performed around that time, demonstrated a small hyperintensity in the spinal cord at the level of the craniocervical junction and a hyperintense signal at the levels of C4–C5 and C5–C6, with discus hernia. Furthermore, the patient had experienced another neurological episode of discrete right-sided hemiparesis of unknown etiology 14 years earlier. Investigation with MRI of the cervical spine at that time showed a myelopathy at C4 and cervical stenosis at the level below. MS had been considered in the differential diagnosis but was not confirmed at that point in time, as investigation of the cerebrospinal fluid did not demonstrate oligoclonal bands. The previously mentioned “stroke” in our patient's medical history had been an interpretation of an episode of right facial palsy, loss of strength in the right arm, diplopia, and tendency to fall to the right side. However, a brain MRI was not performed to confirm the clinically suspected diagnosis of stroke. We believe the previous episodes of neurological dysfunction, including the “possible stroke,” were in fact MS relapses. Indeed, brain MRI showed evidence of inflammatory T2 hyperintense lesions and no lesions compatible with previous stroke(s).

Based on the MRI findings and combined with previous and current clinical neurological symptomatology, the diagnosis of relapsing–remitting MS (RRMS) was made. The patient fulfilled the 2010 McDonald criteria for MS, with clinical episodes providing evidence for dissemination in time and space (20). Brain and cervical spinal cord MRIs were compatible with dissemination in space according to the 2010 McDonald criteria (20).

Transthoracic echocardiogram (TTE) showed almost complete recovery of the systolic left ventricular function. ECG showed normalization of previous changes, and only non-specific ST-T changes were present (Supplementary File 12-lead ECG 4). During her stay at the ICU, there were recurrent episodes of uncontrolled hypertension, perspiration without fever or distress, and repetitive hypercapnic respiratory failure based on central apnea, necessitating intubation with ventilatory support for four times. She was treated with pulsed high-dose intravenous methylprednisolone, plasma exchange, and intravenous immunoglobulins (400 mg/kg) for 5 days. This was followed by treatment with high-dose cyclophosphamide due to lack of clinical response to previous treatments and severity of clinical symptoms.

Three months after admission to the ICU, she was discharged to the neurology ward but readmitted 2 weeks later with recurrence of central hypercapnic respiratory failure due to autonomic dysfunction, probably caused by the brainstem lesion. Again, the episode was accompanied by hypertension (216/110 mmHg) and sinus tachycardia (120 bpm). TTE did not show recurrence of TTS at that time. Because of relapsing episodes of untreatable autonomic dysfunction and the poor prognosis for further recovery, the patient decided to stop supportive treatment, and she died 4 months after initial presentation.

## Discussion

While MS exacerbations and lesions in the medulla oblongata with cardiopulmonary presentations have been described before (21), we present the first case of recurrent TTS leading to a diagnosis of MS in an elderly patient. In older patients, a diagnosis of MS may be challenging because of the atypical age and medical history with cardiovascular risk factors. However, when reassessing the patient history, we identified several episodes compatible with MS relapses over the course of more than 30 years. The age of our patient, 69 years, is comparable with the mean age to develop TTS (1), but older than has been published in the literature (MS patients with TTS had an age range of 14–55 years) (21). Our patient recovered from two episodes of TTS but eventually died after stopping the supportive treatment in the ICU. Several negative prognostic factors were present in our patient. Dyspnea on admission is known to correlate with worse outcome, including in-hospital complications and mortality (22). Neurological disorders, low LVEF on admission, and cardiogenic shock, all present in our case, have been associated with worse outcome (2, 23). The InterTAK registry has shown that physical triggers carry a higher mortality risk than emotional ones (24). Moreover, patients with TTS secondary to neurological diseases had the worst short-term (30 days) prognosis and neurological diseases, as a cause of TTS remained a negative prognostic factor in the long term (5 years) (24).

The cardiovascular comorbidities and the presence of coronary artery disease (CAD) at presentation complicated the diagnosis in this case. However, two points need to be made here. First, TTS can be triggered by acute coronary syndrome (25). Hence, significant CAD does not exclude the diagnosis of TTS, which is also reflected in the International Expert Consensus Document on TTS (26). Our case fulfills the suggested diagnostic criteria for TTS (26). Second, the regional wall abnormalities did not match the location of the CAD in our patient and were consistent with TTS. The recovery of the ventricular function without intervention, after the first episode, supports the diagnosis of TTS in our patient. The normal MIBG scan does not exclude a diagnosis of TTS in our patient, as this scan was performed ~1 week after a TTE demonstrated almost full recovery of TTS, showing only a mild global reduced contractility without regional wall motion disturbances.

While the pathogenesis of TTS remains incompletely understood, the association with emotional or psychological stress factors and neurological diseases preceding the onset of TTS emphasizes the existence of a link between the heart and the brain (26). Indeed, TTS has been associated with a variety of neurological causes, among which subarachnoid hemorrhage, status epilepticus, and seizures have been reported as the strongest associated acute neurological diseases (5, 26–28). Data analysis from the InterTAK registry demonstrated that 4.7% of patients (*N* = 66/1,402) had TTS recurrence with intervals ranging from 30 days to 9.9 years (29). Both neurological and psychiatric disorders were found to be independent predictors of recurrence (29). In 34.8% (*N* = 23/66) patients, the ballooning pattern differed between the first and subsequent presentations (29). All patients with multiple recurrent events had psychiatric or neurological comorbidities (29). Recent research has linked a decreased functioning of brain regions, which are associated with autonomic functions and the occurrence of TTS (1, 26, 30). A recent review of published cases of MS and TTS found that in the majority, a demyelinating lesion located in the medulla oblongata was present (21).

One hypothesis of the pathophysiology of TTS is based on an increase of catecholamines. This can lead, via multiple mechanisms, to direct catecholamine toxicity and adrenoceptor-mediated damage resulting in epicardial and microvascular coronary vasoconstriction and/or spasm and increased cardiac workload. This induces myocardial damage, which has a functional counterpart of transient apical left ventricular ballooning (31, 32). In our patient, increased plasma and 24-h urinary excretion levels of norepinephrine and normetanephrine were detected during the second episode of TTS. Since levels at first presentation were not measured and only measured once during the disease course, it is difficult to draw firm conclusions on the relevance of the catecholamine levels in the development of TTS in our case. A recently published meta-analysis of catecholamine plasma levels in TTS demonstrated that levels of norepinephrine, epinephrine, and dopamine are elevated in TTS, while marked elevation is rare (32). A link to prognosis of TTS remains to be proven.

We hypothesize that in our case, direct suppression of vagal-mediated cardio-inhibition played a role in the development of TTS. Indeed, our patient had a critically located MS lesion in the dorsolateral medulla oblongata involving the nucleus ambiguus, which contains cardio-inhibitory cholinergic preganglionic parasympathetic neurons (33). The role of impairment of vagal nerve fibers in development of TTS and blood pressure dysregulation has been reviewed extensively by Norcliffe-Kaufmann (34).

## Limitations

Several limitations of our case study need to be addressed. Autopsy was not performed, and therefore, the diagnosis of MS was not confirmed postmortem. However, our patient fulfilled McDonald 2010 diagnostic criteria for MS, and other potential causes for the brain lesions were excluded (20). Also, pathological examination of the heart is missing, which could have confirmed the clinical findings of limited or no cardiac ischemia, supporting the diagnosis of TTS. Despite the lack of autopsy material, additional investigations were compatible with a diagnosis of TTS. Moreover, the presence of CAD does not exclude a diagnosis of TTS (26). Finally, while no formal autonomic function tests were performed in our patient due to her clinical condition, the clinical symptoms with profound blood pressure fluctuations, hypothermia, and hypoventilation are suggestive of autonomic dysregulation and compatible with symptoms occurring as a consequence of the lesion in the medulla oblongata.

## Future Directions

This case supports the hypothesis of the link between neurological disease and TTS, especially with involvement of the medulla oblongata. Further research of the pathophysiology of brain stem disease and/or lesions, especially located in the medulla oblongata, and the occurrence of TTS is needed and may provide new insights in the pathophysiology and treatment of this syndrome.

## Conclusions

This case illustrates a link between a single critical demyelinating lesion in the dorsolateral medulla oblongata and TTS. Diagnosis of TTS in combination with signs of autonomic dysfunction warrants thorough investigation of possible underlying or concomitant neurological disease, including MS, even in older patients with cardiovascular risk factors.

## Data Availability Statement

The original contributions presented in the study are included in the article/Supplementary Material, further inquiries can be directed to the corresponding author/s.

## Ethics Statement

Ethical review and approval was not required for this case study on human participants in accordance with the local legislation and institutional requirements. The patient's relative provided their written informed consent to participate in this study. Written informed consent was obtained from the patient's relative for the publication of any potentially identifiable images or data included in this article.

## Author Contributions

FR, PM, and BW conceived the idea for the manuscript. FR and PM drafted the first version. FR and BW drafted the revised manuscript. PM, IB, CF, and BW critically revised the paper for important intellectual content. SN selected the MRI images and provided the description. All authors approved the final version of the manuscript.

## Conflict of Interest

The authors declare that the research was conducted in the absence of any commercial or financial relationships that could be construed as a potential conflict of interest.

## Publisher's Note

All claims expressed in this article are solely those of the authors and do not necessarily represent those of their affiliated organizations, or those of the publisher, the editors and the reviewers. Any product that may be evaluated in this article, or claim that may be made by its manufacturer, is not guaranteed or endorsed by the publisher.

## Supplementary Material

Supplementary Figure 1. 12-lead ECG 1 12-lead ECG on 25 mm/s paper speed and 10 mm/mV y-axis with regular sinus-rhythm at 111 beats per minute (tachycardia). There is slight residual 1 mm ST-elevation in lead II and V4-V6. There is Q-wave formation present on inferior (II, III, and aVF) and anterolateral leads (I, V2-V6) and a small R wave on anterior leads (V1-V6).

Supplementary Figure 2. 12-lead ECG 2 12-lead ECG on 25 mm/s paper speed and 10 mm/mV y-axis with regular sinus-rhythm at 86 beats per minute. There is convex ST elevation, most appreciated in two of the inferior leads (II, aVF) and anterolateral (I, V2-V6). Biphasic T wave formation anterior (V2-V4) and negative T waves in aVL. Q-waves are present inferior (II, III, and aVF), lateral (I, aVL, V5-V6) and anterior (V3-V4).

Supplementary Figure 3. 12-lead ECG 3 12-lead ECG on 25 mm/s paper speed and 10 mm/mV y-axis with regular sinus-rhythm at 78 beats per minute. Negative T waves are present inferior (II, III, and aVF), lateral (I, V5-V6) and anterior (V2-V4). ST-segment in aVL is flattened. There is only a small Q wave remaining in the lateral (I, aVL, V5, V6) leads.

Supplementary Figure 4. 12-lead ECG 4 12-lead ECG on 25 mm/s paper speed and 10 mm/mV y-axis with regular sinus-rhythm at 124 beats per minute (tachycardia). There is recuperation of the earlier mentioned ECG-changes with no more pathological ST-segments, T-waves, or Q-waves present. There is a diffuse flattened ST-segment.
