# Supplementary figures and images for: The Brain–Heart Link: A Case Report of a Critically Located Multiple Sclerosis Lesion in the Brainstem Leading to Recurrent Takotsubo Syndrome
Source: Front Cardiovasc Med. 2021 Aug 9;8:674118. doi: 10.3389/fcvm.2021.674118 (PMC8381246; doi:10.3389/fcvm.2021.674118)

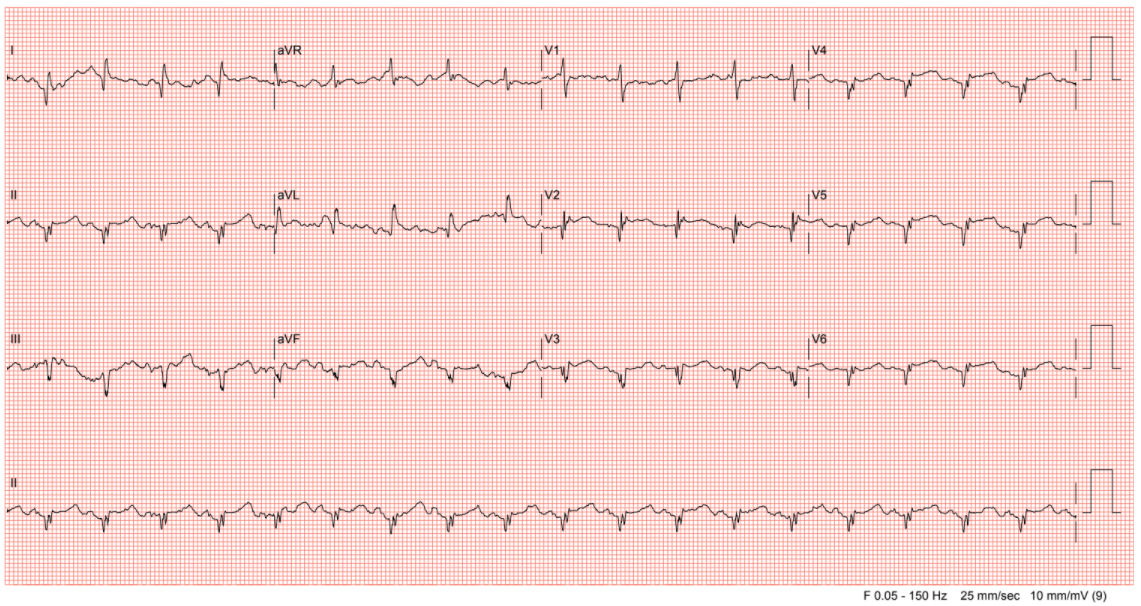

Supplement: Supplementary Figure 1. 12-lead ECG 1 — 12-lead ECG on 25 mm/s paper speed and 10 mm/mV y-axis with regular sinus-rhythm at 111 beats per minute (tachycardia). There is slight residual 1 mm ST-elevation in lead II and V4-V6. There is Q-wave formation present on inferior (II, III, and aVF) and anterolateral leads (I, V2-V6) and a small R wave on anterior leads (V1-V6). [file Image_1.JPEG]

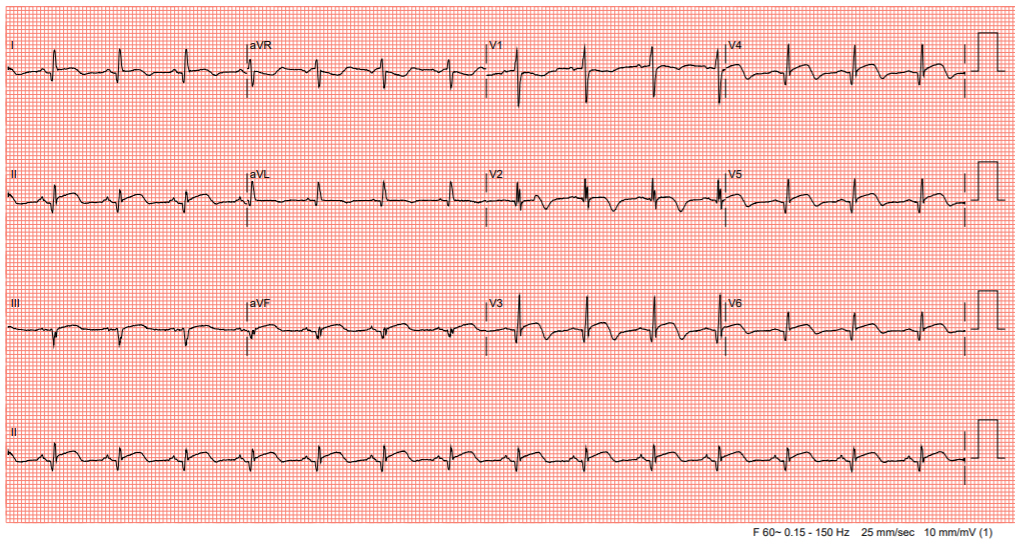

Supplement: Supplementary Figure 2. 12-lead ECG 2 — 12-lead ECG on 25 mm/s paper speed and 10 mm/mV y-axis with regular sinus-rhythm at 86 beats per minute. There is convex ST elevation, most appreciated in two of the inferior leads (II, aVF) and anterolateral (I, V2-V6). Biphasic T wave formation anterior (V2-V4) and negative T waves in aVL. Q-waves are present inferior (II, III, and aVF), lateral (I, aVL, V5-V6) and anterior (V3-V4). [file Image_2.JPEG]

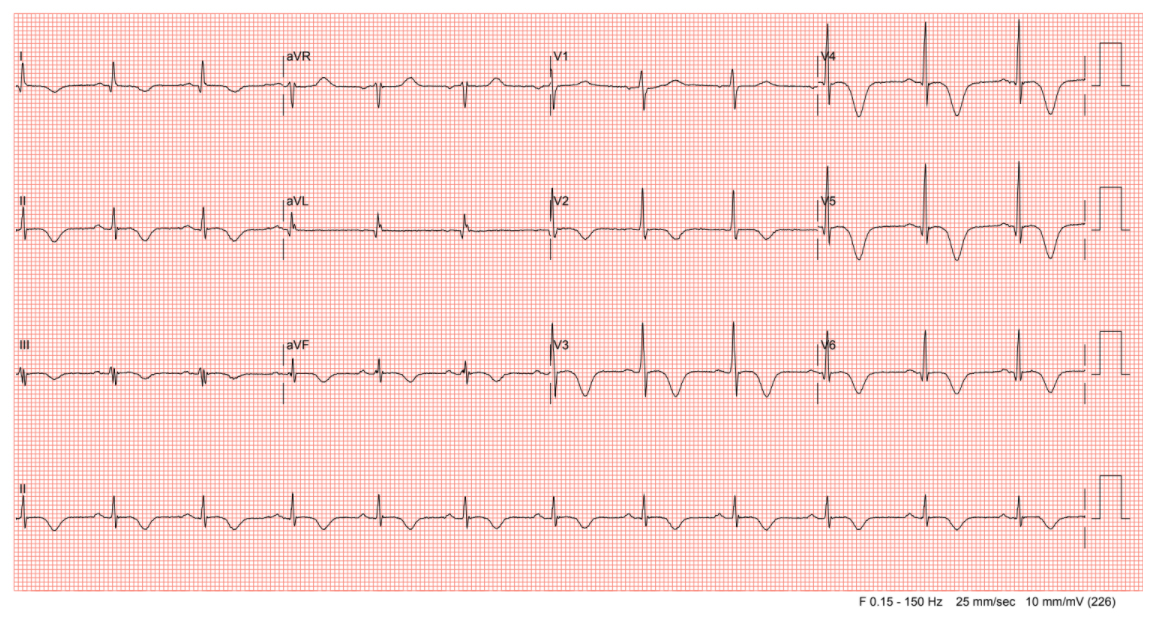

Supplement: Supplementary Figure 3. 12-lead ECG 3 — 12-lead ECG on 25 mm/s paper speed and 10 mm/mV y-axis with regular sinus-rhythm at 78 beats per minute. Negative T waves are present inferior (II, III, and aVF), lateral (I, V5-V6) and anterior (V2-V4). ST-segment in aVL is flattened. There is only a small Q wave remaining in the lateral (I, aVL, V5, V6) leads. [file Image_3.JPEG]

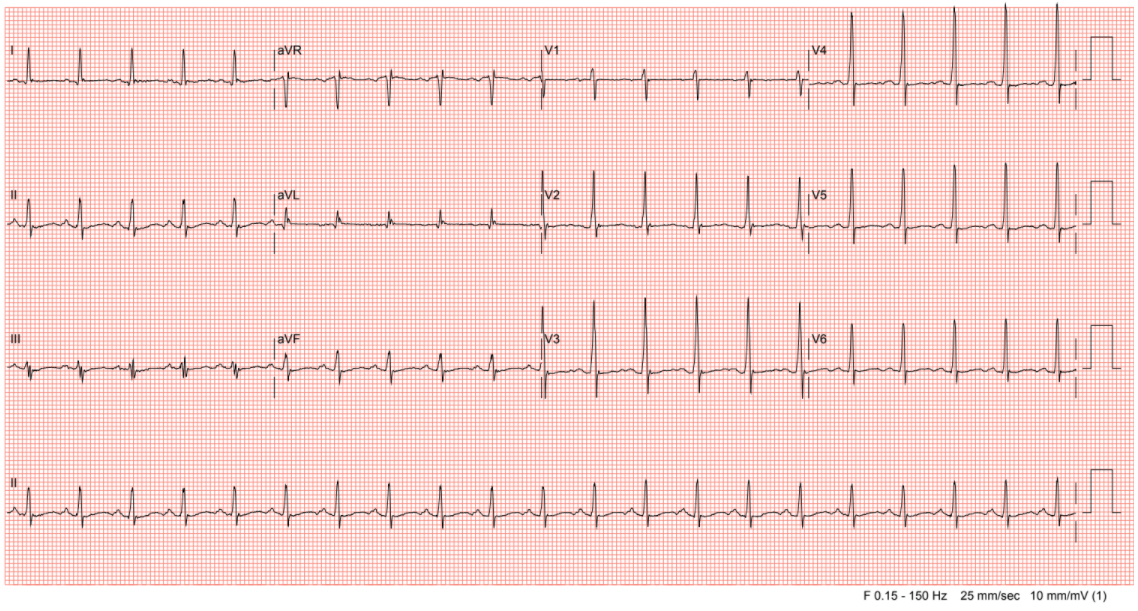

Supplement: Supplementary Figure 4. 12-lead ECG 4 — 12-lead ECG on 25 mm/s paper speed and 10 mm/mV y-axis with regular sinus-rhythm at 124 beats per minute (tachycardia). There is recuperation of the earlier mentioned ECG-changes with no more pathological ST-segments, T-waves, or Q-waves present. There is a diffuse flattened ST-segment. [file Image_4.JPEG]
